# Work Hours and Self rated Health of Hospital Doctors in Norway and Germany. A comparative study on national samples

**DOI:** 10.1186/1472-6963-11-40

**Published:** 2011-02-21

**Authors:** Judith Rosta, Olaf G Aasland

**Affiliations:** 1 The Research Institute of the Norwegian Medical Association, P.O.B 1152 Sentrum 0107 Oslo, Norway; 2 The Research Institute of the Norwegian Medical Association, P.O.B 1152 Sentrum 0107 Oslo, Norway; 3 Institute of Health and Society, Department of Health Management and Health Economics, University of Oslo, Norway

## Abstract

**Background:**

The relationship between extended work hours and health is well documented among hospital doctors, but the effect of national differences in work hours on health is unexplored. The study examines the relationship between work hours and self rated health in two national samples of hospital doctors.

**Methods:**

The study population consisted of representative samples of 1,260 German and 562 Norwegian hospital doctors aged 25-65 years (N = 1,822) who received postal questionnaires in 2006 (Germany) and 2008 (Norway). The questionnaires contained items on demography, work hours (number of hours per workday and on-call per month) and self rated subjective health on a five point scale - dichotomized into "good" (above average) and "average or below".

**Results:**

Compared to Norway, a significantly higher proportion of German doctors exceeded a 9 hour work day (58.8% vs. 26.7%) and 60 hours on-call per month (63.4% vs. 18.3%). Every third (32.2%) hospital doctor in Germany worked more than this, while this pattern was rare in Norway (2.9%). In a logistic regression model, working in Norway (OR 4.17; 95% CI 3.02-5.73), age 25-44 years (OR 1.66; 95% CI 1.29-2.14) and not exceeding 9 hour work day and 60 hours on-call per month (OR 1.35; 95% CI 1.03-1.77) were all independent significant predictors of good self reported health.

**Conclusion:**

A lower percentage of German hospital doctors reported self rated health as "good", which is partly explained by the differences in work time pattern. Initiatives to increase doctors' control over their work time are recommended.

## Background

The detrimental effect of long work hours on health in different occupational groups, including the medical profession, is well documented, [1]. It is also well known that working long hours, as a result of extended days and on-call duties, is common among many hospital doctors in Europe. Although the Working Time Directives of the European Union [2,3] and rulings of the European Court of Justice [4,5] limit the work hours of doctors in the member countries, there are large national variations in the actual work time burden of hospital doctors in Europe [6]. The possible association between international differences in actual work hours and the general health status of hospital doctors is of interest.

A comparison of previous studies is limited by methodological differences regarding data collection, sample characteristics and measurements. However, there is evidence for a considerable difference in work time burden for hospital doctors in two European countries - Norway and Germany. More leisure time and shorter and more regulated work hours in Norwegian hospitals have been a main motive for the migration of German hospital doctors to Norway [7]. Hospital doctors in Germany report significantly lower job satisfaction, compared with their colleagues in Norway, and the largest difference was observed on satisfaction with work hours as one of ten components of the job satisfaction scale [8]. In this paper we look into the differences in doctors' actual work hours in Norwegian and German hospitals, and whether this difference is associated with self rated health.

It is feasible to perform reliable and comparable analyses in these two countries. The general health status of the populations in Norway and Germany, expressed by life expectancy at birth and estimated percentage of life lived in good health or free of disability are similar [9].

The effort-recovery model [10] explains the relationship between long work hours and poor health. It implies that long hours can lead to insufficient recovery, which in turn may cause various health problems [1]. Other theoretical models have assumed that the number of hours worked is directly related to stress [11], which may challenge the doctors' mental and physical health. Excessive work hours and insufficient rest periods are commonly known to be exhausting. Consequently, previous investigations have tended to concentrate on the effect of work hours on mental health such as fatigue, mood changes, sleep disturbance and burnout [12]. Most studies have focused on specific positions or specialties, while little attention has been given to the whole group of hospital doctors [13,14].

The aim of this study is to examine and compare the associations between actual work hours and self rated health in national samples of Norwegian and German hospital doctors. We expect to find that hospital doctors in Germany report longer work hours and poorer health than their colleagues in Norway.

To our knowledge, no comparative study like this has been done; hence this study may be of importance in the present discussion on doctors' work hours [6,15] and health [14,16].

## Methods

### Data collection and sample

In Germany a 12 page postal questionnaire was sent in September/October 2006 from the German Hospital Institute to 3,295 hospital doctors, with no reminders. In Norway a 14 page postal questionnaire was sent in October and November 2008 to 1,650 doctors of all kinds, with one reminder. Both The German Hospital Institute and The Research Institute of The Norwegian Medical Association are independent research institutes with experience in surveys on doctors' health and work conditions. The response rates were 58.2% (1,917/3,295) in Germany and 65.0% (1,072/1,650) in Norway, of which 592 were hospital doctors. Age between 25 and 65 years and working in a hospital setting with a traditional work pattern - day time work usually combined with on-call duties - were inclusion criteria. The final sample comprised 1,822 respondents, 1,260 in Germany and 562 in Norway.

### Questionnaire and measurement

Both the German and the Norwegian questionnaire included a question on the average number of work hours per day: *"On an average work day, how many hours do you work (including overtime, excluding on-call duties)"*. In addition, the average number of hours on-call per month was recorded, in Norway with the question: *"In an average month, about how many hours do you have on-call duties?"*, in Germany with a similar question: *"In an average month, about how many on-call duties do you have on weekday and weekend? About how many hours are you on-call duty on a weekday and weekend*?" The standard full time workweek is between 38-40 hours in Norway and 40-42 hours in Germany [17,18], and almost all our respondents worked at least this much. Only doctors working full time were included in the study.

Work hours of most hospital doctors in Norway, Germany and in other countries consist of hours at work days and on call duties. Work hours among hospital doctors can be measured by using a composite index of hours at work day and on-call duties [14,19]. Our hypothesis is that German hospital doctors report longer work hours and poorer health than their Norwegian colleagues. For the purpose of this study we made a distinction between the doctors who worked both more than 9 hours per day and more than 60 hours on-call per month, and those who did not. Since very few Norwegian doctors meet these criteria we denote not having this pattern of long hours for the "Norwegian work time pattern".

Health was measured by a single question: *"In general, would you say your health is (G: Wie würden Sie Ihren gegenwärtigen Gesundheitszustand beschrieben? N: Stort sett, vil du si at din helse er:) *with response alternatives in Germany *very good (1: sehr gut)*, *good (2: gut), average (3: zufriedenstellend*, synonym for *durchschnittlich *[20]), *less good (4: weniger gut) *, and *poor (5: schlecht) *and in Norway *good (1: god), fairly good (2: nokså god), average (3: middels), rather poor (4: nokså dårlig)*, and *poor (5: dårlig)*. The wording of the two highest response levels differed in the two countries; "very good" and "good" in Germany and "good" and "fairly good" in Norway. However, the middle (average) level is the same, as are the levels below. Hence we dichotomized the original five response levels into "good" (above average; categories 1, 2) and "average or below" (categories 3-5).

The question on self rated health is thoroughly validated and widely used in Norwegian [21], German and other surveys [22]. It is also considered to be a good indicator of mortality risk, morbidity, and general health status [21-23].

### Analyses

We compared proportions by Pearson's Chi-square test and interval variables (age) by calculating 95% confidence intervals. Logistic regression analyses were used to assess the simultaneous effects of workplace country, age, gender and work hours on self rated health. Units with missing items were excluded. SPSS, version 17.0 was used for the analyses.

## Results

### Sample characteristics

The gender distribution was similar in Germany and Norway, with 62.1% (783/1260) and 58.2% (327/560) males respectively. The German doctors were significantly younger, with a mean age of 42.7 (95% CI 42.1 to 43.3) vs. 48.6 (47.5 to 49.7) years for males, and 38.6 (37.9 to 39.4) vs. 42.7 (41.5 to 43.8) years for females.

### Work hours and health

Work hours and self rated general health are shown in Table 1. The work hours per day and on-call duties per month were significantly lower among both female and male hospital doctors in Norway than in Germany. A considerable lower proportion of Norwegian doctors exceeded a 9 hours' work day plus 60 hours on-call per month.

**Table 1:** Work time and self rated health of hospital doctors in Norway and Germany, aged 25-65 years and employed in full time. Data are % (n) of respondents.

	All hospital doctors		Male hospital doctors		Female hospital doctors	
**Variable**	**Norway % (n)**	**Germany % (n)**	**Norway % (n)**	**Germany % (n)**	**Norway % (n)**	**Germany % (n)**

**Hours per work day**						
≤ 8	31.3* (157)	9.0* (99)	23.3* (70)	8.8* (67)	43.2* (86)	9.6* (32)
8.1-9.0	42.0* (211)	32.1* (352)	42.5* (128)	29.3* (223)	41.2 (82)	38.5 (129)
> 9	26.7* (134)	58.8 *(644)	34.2* (103)	61.8* (470)	15.6* (31)	51.9 *(174)

**Hours on-call duty per months**						
0	27.6 (134)	30.6 (316)	29.1 (85)	34.4 (245)	25.5 (49)	22.1 (71)
1-60	54.1* (263)	6.0* (62)	58.2* (170)	5.4* (38)	47.4* (91)	7.5* (24)
> 60	18.3* (89)	63.4* (656)	12.6* (37)	60.3* (430)	27.1* (52)	70.4* (226)

**Norwegian work time pattern **(not having the combination of >9 hours work day and >60 hours on-call per month)						
Excl. doctors with "0" on-call duties	96.1* (332)	53.9*(386)	96.0* (194)	52.1* (241)	95.8* (135)	57.3* (139)
Incl. doctors with "0" on call-duty	97.1* (465)	67.8* (696)	97.2* (279)	68.5* (486)	96.8* (184)	66.5* (210)

**Self rated health as good**	88.1* (450)	63.3* (698)	88.3* (272)	62.9* (480)	88.1* (177)	64.1* (218)

* p < 0.0001, differences between countries using Pearson' Chi-square test

In both countries, male doctors worked significantly longer days and female doctors more hours on-call. However, we found no sex differences in the prevalence of Norwegian work time pattern (data not shown).

The majority of the doctors in both countries reported good health, but this proportion was significantly lower in Germany (Table 1). There were no gender differences in self rated health in either country (data not shown).

In a logistic regression model (Table 2, Model I) the simultaneous effect of sex, age and work country on self rated health was explored. The model fit the data fairly well (p = .358, Hosmer-Lemeshow test). When "Norwegian work time pattern" was included as predictor (Model II), there was a moderate decrease in -2 Log likelihood from 1681 to 1677, suggesting an improvement in model fit (to p = .498, Hosmer Lemeshow).

**Table 2:** Logistic regressions with good self rated health as response variable, without (Model I) and with (Model II) the Norwegian work time pattern. 1 503 full time hospital doctors in Germany and Norway.

	Model I			Model II		
		
Predictor	OR	95% C.I. for OR	p	OR	95% C.I. for OR	p
Male	1.06	.82-1.37	.642	1.07	0.83 - 1.38	0.625
Age 25 to 44	1.59	1.24-2.04	<.001	1.66	1.29 - 2.14	<.001
Working in Norway	4.50	3.30-6.14	<.001	4.17	3.02 - 5.73	<.001
Norwegian work time pattern (‡)				1.35	1.03-1.77	0.031
	
Hosmer-Lemeshow test		χ^2 ^= 5.5, df 5	.358		χ^2 ^= 6.4, df 7	.498

(‡) Not having the combination of working more than 9 hours a day and more than 60 hours a month on-call

## Discussion

The present study shows how self rated health is associated with hospital doctors' work hours in Norway and Germany. German doctors work considerably longer hours and report significantly lower rates of good self rated health than their Norwegian colleagues.

The Norwegian work time pattern (Table 2) was a significant predictor of good self rated health. This can partly be explained by more recovery time [1,10] and less strain related to long work hours [11]. In a logistic regression, the effect of working in Norway was a stronger independent predictor of good self rated health than following the Norwegian work time pattern. This suggests that cultural factors other than the actual work time pattern account for part of the observed difference in self reported health.

Hospital doctors' work conditions are strongly associated with the work organisation [24] and the national directives [15]. Thus, the national regulations of work conditions - all aspects of work life including salary, control over clinical work and professional autonomy, collegial support and work time - may impact on the doctors' health.

There are considerable differences in work conditions for doctors in the two countries. A recent study on job satisfaction of Norwegian and German hospital doctors shows that Norwegian doctors enjoy a higher level of job satisfaction, suggesting a better work atmosphere in Norwegian hospitals, with lower physical burden, better collegial environment, more professional autonomy, more control over clinical work and shorter work hours [8]. That job satisfaction and other work conditions are determinants of health are well documented [25,26]. In Germany, several regulations and restrictions on doctors' remuneration and workload have been implemented during the last few years. The workload, expressed by increasing patient throughput and a corresponding reduction in the average duration of hospital stay, has increased. The situation is aggravated by understaffing and increasing migration of German doctors to other countries, often motivated by unacceptable work conditions [27]. In 2006 work hours increased from 38.5 to 40 or 42 hours per week for most of German hospital doctors without a corresponding increase in salary [18]. Working overtime - usually uncompensated - is considered the norm in German hospitals [28]. In Norway, regular weekly hours for hospital doctors have remained stable at 38 to 40 for the last decade [17] with a steady growth in salary [29]. Norwegian hospital doctors also have a lower workload in terms of number of hospital dismissals and more practising doctors per capita [30].

Another cultural difference might lie in the adherence to mandatory regulations of hospital doctors' work time. According to a member survey of the German doctors union [28] and a report of the Norwegian Medical Association [31], the majority of doctors in German hospitals (59%) complained about the renege on stipulated maximum weekly work hours, while only 30% of the Norwegian hospital doctors reported a pressure from the hospital administrations to deviate from the work time agreements. Respect for work time regulations and a good balance between professional and private life seem to be important cultural values in Norway. In the most recent European Working Conditions Survey [32], Norway was found to have the second-lowest average weekly work time, and the lowest percentage among European countries of employees with a weekly work time over 48 hours.

According to the job demand-control model of Karasek and Theorell [33], high job demands (workload) in combination with low job control (autonomy, decision latitude) may have negative health effects, and work overload has been shown to be a significant stressor among doctors [11]. A Norwegian study documents that stress among doctors increases with increasing voluntary or involuntary overtime [34]. A recent survey of Dutch full-time employees concludes that involuntary overtime without reward represents a threat to the workers' health [35].

Thus, the significantly lower percentage of doctors in Germany with good self reported health could be ascribed not only to the higher amount of work hours on weekdays and on call duties, but also to negative aspects of the work organization such as higher work load, less autonomy in job-related decisions combined with less control over work hours and higher demand for uncompensated overtime. Unfortunately we did not have comparable data on these worklife aspects for the present study.

In terms of health care policy, better work time control could be the first step to improve doctors' health. Work time reduction and control have traditionally been seen as a feature of health care in the European work time regulations [2-5]. Good professional climate, high professional autonomy and monetary recognition of clinical work are also essential [11,33-35]. The health of the doctors is an important public health issue with direct bearing on the quality and stability of health care systems, as well as on the doctors' well-being [1,13,14,16].

### Strengths and limitations

The strength of this study lies first and foremost in the comparative and representative datasets, making the results generalisable to the entire population of hospital doctors in Germany and Norway. The high validity of the self rated health question [22,23], similarities in measurement methods, and comparable elements of work hours are also strengths of the study.

One limitation is clearly a possible cultural difference in health perception. According to the "World Value Survey" [36] 79.5% of Norwegians rate their subjective health as "good or very good", compared to 71.6% in Germany, indicating either a difference in health perception or an actual difference in population health. The latter is more or less ruled out by data from the "Atlas of the Health in Europe" [9] where the general health status expressed by estimated percentage of life lived in good health or free of disability is similar in Norway (male: 92.2%, female: 90.1%) and Germany (male: 92.1%, female: 90.7%). Furthermore, in a recent Finnish study on self rated health [23] it is argued that doctors "probably share a fairly similar general understanding of what constitutes health and what information is essential to describing it", indicating that the intercultural reliability of our health measure should be sufficient, particularly since our respondents are all doctors.

The fact that the two highest response levels of the health question had different wordings is of concern. However, since the "average" and the lower levels were identical, the dichotomization of this measure should make the two samples directly comparable.

One might speculate whether the two year time difference between the surveys (2006 in Germany and 2008 in Norway) may affect the results, but this does not seem to be the case. Between 2006 and 2008, the regulations of contracted weekly hours (N: 38-40 hours; G: 38.5-42 hours) and the maximum weekly hours including on-call of hospital doctors (N: 60 hours; G: 66 hours) remained unchanged [17,18,37,38]. According to a recent analysis, the satisfaction with work time among Norwegian hospital doctors was found to be stable from 2000 to 2006 [39]. In Germany, reports about poor working conditions, including low income, high workload and long work hours among hospital doctors continued from 2006 to 2008 [18].

A further limitation is the relatively low response rates. This may reflect a limited willingness to participate in surveys compared with other Europeans [40]. Another reason for non-response could be that the doctors did not find time to complete the questionnaire. Nevertheless, it should be noted that despite the fact that no reminder was sent in Germany, response rates of 58.2% and 65% are better than in many other doctor surveys [19].

The intensity of on-call duties from home was not measured in Germany. Many doctors perform on-call duties from home up to every other day, as "backup cover". It should also be taken into account that scientific and administrative tasks are often carried out at home after regular work hours. These factors would increase the actual work time still further.

Differences in specialty patterns might explain some of our findings. In Norway, 14.5% of the respondents worked in the surgical domain, 34.0% in internal medicine and 51.5% in other specialties. In Germany the respective proportions were 29.7%, 29.1% and 41.2%. In Germany daily work hours were identical (median 10 hours) and monthly hours on-call higher among surgeons (median 128 hours) than in internal medicine (median 112 hours). In Norway surgeons and internal medicine doctors worked similar hours, median 9 hours per day and 19-20 hours on call per month. However, the inclusion of specialty as categorical variable in our logistic model (table 2) did not make any significant difference.

In our final model (table 2) we have included work hours, age, sex and workplace country as possible predictors of good self reported health. It is likely that also other variables such as coping, other workplace hazards or local regulations affect the relationship between work hours and health [1,35], but such data have not been available for this study.

Furthermore, the study only includes doctors who are currently working in hospitals, and not those who have already left their jobs due to excessive demands or ill health. At present, there is an increasing influx of German doctors to other professions or to other countries, including Norway - usually driven by demanding work schedules and excessive work hours [7,27]. Therefore, it would be interesting to collect data from the hospital doctors in Germany who have moved to Norway, and compare with the doctors still working in German hospitals.

## Conclusion

The current study contributes to the international literature on work time of hospital doctors in particular by documenting an association between work hour patterns and self rated health. A lower percentage of German hospital doctors reported self rated health as "good", and controlled for other possible cultural differences, the work time pattern was a significant predictor of self rated health. Improved work organisation, in the form of reduced work hours, as well as better control over own work time, preferably combined with lower work load and reward for overwork are recommended strategies to improve doctors' health.

## Competing interest

We declare that we have no conflicts of interest.

## Authors' contributions

Both authors have many years of experience in statistics and survey methods, and contributed equally to analysing the data and writing the article.

## Pre-publication history

The pre-publication history for this paper can be accessed here:

http://www.biomedcentral.com/1472-6963/11/40/prepub
